# Faith-Based Coping Among Arabic-Speaking Refugees Seeking Mental Health Services in Berlin, Germany: An Exploratory Qualitative Study

**DOI:** 10.3389/fpsyt.2021.595979

**Published:** 2021-02-01

**Authors:** Diana Rayes, Carine Karnouk, Dana Churbaji, Lena Walther, Malek Bajbouj

**Affiliations:** ^1^Department of Psychiatry and Psychotherapy, Charité University Medicine Berlin, Berlin, Germany; ^2^Department of International Health, Johns Hopkins Bloomberg School of Public Health, Baltimore, MD, United States; ^3^Institute of Psychology, University of Münster, Münster, Germany

**Keywords:** asylum-seekers, refugees, Muslim, faith-based coping, integration, mental health, Germany

## Abstract

**Background:** The benefits of faith-based coping or using religious and spiritual beliefs as a stabilizing force for interpreting stressful or distressing events are largely unexplored among the exodus of Arabic-speaking refugee populations from Muslim-majority countries, particularly those resettled in Europe. The present study aimed to explore the manifestation of faith-based coping strategies among Arabic-speaking refugee adults seeking mental healthcare services in Berlin, Germany and explore how favorable faith-based coping strategies can be optimized from a mental health service-delivery and broader integration perspective.

**Methods:** A total of 17 qualitative interviews were conducted with Arabic-speaking refugee adults (six females, 11 males) seeking mental health services at the Charité Universitaetsmedizin in Berlin. Research questions aimed to solicit comprehensive perspectives from refugee adults on their mental health, with an emphasis on faith-based coping, and how this facilitated or impeded their integration into German society. Interview transcripts were translated to English from Arabic and analyzed using MAXQDA (2018) to highlight thematic patterns using a grounded theory approach.

**Results:** Findings were structured into four themes, including: (I) faith-based coping methods during flight, (II) changes in faith practices upon arrival, (III) faith-based coping methods to address distress during integration, and (IV) advice for German mental healthcare providers. Participants who demonstrated a stronger commitment to faith were more likely to utilize faith-based coping strategies when seeking mental health services and facing the challenges of displacement and integration. Examples of faith-based coping included prayer, supplication, reciting scripture, and seeking help from a local religious leader.

**Conclusion:** The findings suggest how faith and faith practices play a significant role in the mental health and integration of refugee populations in Germany and provide insight on how mental healthcare can be delivered in a culturally-sensitive manner, providing alternatives to the social, cultural, and linguistic barriers posed by the German health system. These findings are particularly relevant for mental health professionals, non-governmental organizations, and humanitarian aid agencies providing mental healthcare to Arabic-speaking populations recently resettled in Western contexts.

## Introduction

As host countries continue to grapple with how to best integrate recently arrived refugees and asylum-seekers into their societies, the influence of faith, including religious or spiritual beliefs and practices, on refugee mental health and well-being remain largely unexplored. Refugees fleeing conflict arrive in foreign countries having experienced the effects of war, shock, upheaval, and the psychological burden of their journeys (1). Studies in Germany suggest that over 40% of refugees and asylum-seekers who have arrived since 2013 show signs of a mental disorder, a quarter of them with diagnosable post-traumatic stress disorder, anxiety, or depression (2–5). This distress is often exacerbated upon arriving to a new host country given various social, economic, and legal barriers imposed on refugees and asylum-seekers (5, 6). Recent studies also report high prevalence rates of up to 75% of mental distress among Syrian refugees resettled in Germany and an increased risk among refugees for developing a severe mental illness in comparison to the host population (7, 8). Language barriers, culture shock, and lack of economic opportunities (9) further discourage refugee populations from engaging with host communities and therefore, delay or inhibit successful integration.

Among the many European countries hosting refugees, Germany has played a vital role in the future of many – receiving nearly one million refugees from Syria, Afghanistan, and Iraq in 2015 alone (10). Since then, Germany has seen the arrival of more refugees and asylum-seekers than any other European country (11). The large influx of refugees to Europe from Muslim-majority countries has inevitably led to a sharp rise in the number of Muslims in Germany (12). Predictions by the Pew Research Center indicate that even with no future net migration, Muslims in Germany will represent 9% of the population by 2050 (13). The sharp rise in numbers of Muslims in Germany has inevitably led to a significant shift in the sociopolitical landscape within Germany and in Europe. This has partly led to the growing influence of populist and nationalist groups with anti-immigration policy agendas, provoking fear of the Muslim threat to Germany's social and religious cohesion (14–16). In addition, discrimination and stereotyping of migrant populations, particularly those from Muslim-majority countries, is often propagated by negative media coverage and misinformation campaigns by the same groups and can lead to feelings of discrimination or isolation (9, 17). This can potentially exacerbate psychological symptoms, leading to isolation and alienation, and further complicate the integration process for refugee communities, particularly those from Muslim-majority countries (18).

More recently, there has been more emphasis on the provision of culturally-sensitive mental health services to refugees and asylum-seekers, especially those fleeing from conflict or those who have experienced political or religious persecution in their country of origin (19). This includes the adaptation of existing mental health and psychosocial support services to be more accessible in different languages and ensure that mental health professionals are aware of various social norms and dynamics that exist within a particular cultural group (20). Coping methods, or efforts made by individuals to manage or overcome their psychosocial distress, can also vary across different cultures. For example, refugee populations usually rely on a social pyramid of family and close friends for support – one that might not exist anymore due to displacement, separation, and the loss of loved ones during conflict (21). With this knowledge, mental health professionals can help their refugee clients identify other forms of social support, including cultural, religious, or diaspora networks, which can help newcomers navigate their new environment and cope with the sudden changes following displacement (20, 22).

Within the umbrella of providing culturally-sensitive mental healthcare, there is a small, yet growing body of evidence on the benefits of faith-based coping, or using religious or spiritual beliefs as a stabilizing force for interpreting traumatic events (23, 24). For example, a review published by UNHCR emphasized the diversity of faith-based coping methods utilized by Syrian refugees from various faiths suffering from mental health problems (20). This includes reading Quranic or verses from scripture, making prayers or supplication, seeking treatment from a religious cleric or traditional healer to keep away *jinn* or evil, or visiting holy sites or completing *hajj* or Islamic pilgrimage. Similar coping methods have been demonstrated by studies regarding Syrian refugee adults resettled in the United States (25) and Somali refugee women in Australia, who reported that daily prayers required by their Islamic faith to be a source of comfort and solace during bouts of depression and loneliness in their new home (26).

Despite the significant implications of these findings for mental health policy and practice, faith-based coping strategies among refugee populations seeking specialized mental health services remain largely unexplored, particularly for those living in Germany and in Europe more broadly. The objectives of this qualitative study were to explore the manifestation of faith-based coping strategies among Arabic-speaking refugee adults seeking mental healthcare services in Berlin, Germany and explore how favorable faith-based coping strategies can be optimized from a mental health service-delivery and broader integration perspective.

## Materials and Methods

### Study Sample

This research was a sub-study of the Mental Health in Refugees and Asylum-Seekers (MEHIRA) project, led by the Charité Universitaetsmedizin Berlin (19, 27). The MEHIRA project is a multi-center randomized controlled trial aimed to investigate the effects of a stepped and collaborative care model (SCCM) for refugee and asylum-seekers suffering from mental health issues in Germany. The study aimed to explore how mental health care can be delivered in a culturally-sensitive manner. This was done by exploring alternatives to the social, cultural, and linguistic barriers posed by the German health system by providing healthcare in the same language as the client, or by a healthcare provider from the same cultural background. Study participants were recruited from the larger MEHIRA study sample, which included adults who were (i) between the age of 18 and 65 (ii) demonstrated no symptoms of neurodegenerative disorder, psychotic disorder, or suicidal ideation (iii) had refugee or asylum-seeker status in Germany (iv) spoke either Arabic or Farsi.

From among the MEHIRA participants, this study sample was limited to Arabic-speaking refugee and asylum-seeker adults seeking care at the Central Clearing Clinic sponsored by the Charité Universitaetsmedizin Berlin. After completing the initial MEHIRA baseline data collection process, including demographics and a number of questionnaires to assess overall psychological well-being (determined via a score of ≥12 on items 1–14 or ≥5 item 15 in the Refugee Health Screener-15, and “several days” or higher in a minimum of three responses in the Patient Health Questionnaire-9), participants were invited to take part in an anonymous interview designed to further solicit their perspectives regarding the importance of their faith to promote well-being and prevent mental illness.

Participants were purposively sampled from among the MEHIRA study population to include a variety of age and gender groups with demonstrated interest in participating in the qualitative study following their baseline assessment. For more information, please refer to the complete MEHIRA study protocol available in (19).

### Data Collection

A semi-structured interview guide (Appendix A) with 19 questions was designed using a grounded theory approach to contextualize questions of mental health and faith-based coping within a comprehensive backdrop of the participant's lived experience (28). This included questions regarding the nature of the war and conflict they fled back home, the experience of their displacement journey, and current challenges faced or experienced following their arrival to Germany in order to illustrate the chronology of mental health symptoms or illness. Supplementary Information regarding aspects of their personal lives, including their family, upbringing, traditions, and cultural practices were also included to elicit a narratives (28). To explore links between mental health, concepts of the self, and faith, questions regarding general coping methods and religious background were adapted from the HOPE Approach to Spiritual Assessment. This included questions about general sources of hope, meaning, comfort, and peace, as well as standard questions regarding the importance of organized religion in the lives of participants and extent of practices that are helpful to the participant (29).

Informed consent was provided by participants before initiating data collection. All interviews took place in a private setting within a mental health clinic in central Berlin between December 2018 and April 2019. Interviews were audio-recorded following the consent of the participants. All interviews were conducted in Arabic by a native Arabic speaker with a psychology background and public health research training (DR). Interviews were simultaneously transcribed and translated to English. *In vivo* codes, including Arabic terms and phrases used to describe culturally specific symptoms or methods of coping were transliterated to English for later inclusion in the results. On average, interviews lasted 36.5 min.

### Qualitative Analysis

The interviews were anonymized, transcribed, and entered to MAXQDA (20.0.8) in English to code and categorize the data into relevant themes using a grounded theory approach (28). Based on this framework, line-by-line coding by DR (also the primary interviewer) was completed in order to comprehensively reexamine the data collected. Codes were then initially organized by topics listed in the interview guide, including challenges, general coping methods, examples of faith-based coping, to facilitate the coding of complex perspectives shared by participants regarding mental health, concepts of the self (including experience of displacement), and faith. Code categories were later expanded based on emerging ideas that were compiled at the end of each interview in order to explore unexpected themes or corroborate certain ideas or responses shared by other participants in subsequent interviews. Interpretation of emerging ideas was triangulated among three of the authors to ensure accuracy. This included codes regarding coping strategies before, during, and after displacement, seeking mental health support from a spiritual leader, impact of integration on faith practices, and advice to mental health professionals.

For the analysis and compilation of themes, a top-down approach was used for targeted interview questions (such as “For some people, their religious or spiritual beliefs act as a source of comfort and strength in dealing with life's ups and downs; is this true for you? If yes, how? If no, was it ever?”) to develop concise, yet comprehensive categories. This led to the development of themes regarding faith-based coping methods utilized before, during, and after displacement, as well as advice for German mental health providers. For more general interview questions (such as “What are your sources of hope, strength, comfort, and peace?”), a bottom-up analysis approach was used to develop important themes based on cultural and religious coping methods demonstrated across the study population, including changes in faith practices upon arrival and integration.

### Ethical Approval

The study was conducted as a part of the larger MEHIRA project, which was approved by the Ethical Committee of the Charité Universitaetsmedizin Berlin. The study was registered in ClinicalTrials.gov (registration number: NCT03109028; registration date 11.04.2017). As exhibiting symptoms of depression or psychological distress was an inclusion criterion for this study, particular approaches were taken to ensure the comfort of the participant before, during, and after the interview. This included taking note of the general affect of the participant throughout the interview, including tone of voice, bodily gestures, and facial expressions, in order to take any potential steps to stop or halt the interview if the participant became upset or uncomfortable. Before the interview, it was noted whether or not the participant had an appointment in the clinic before or after the interview in order to prevent any delays or interview fatigue. Referrals to mental health professionals working in the clinic were available for support and supervision in the event that it was needed.

## Results

### Participants

A total of 17 participants (11 male; six female) were interviewed for the study (see Table 1). Participants were between the ages of 22 and 47 years old, with an average age of 34.7 (see Table 2). The majority of participants were originally from Syria (*N* = 12), followed by Iraq (*N* = 5). A total of 6 participants were married, five were divorced or separated, five were single, and one was widowed. Nine participants had at least one child. On average, participants had been in Germany for 2 years and 3 months (ranging between 8 and 47 months) and had completed 10.4 years of schooling. Reasons for migration varied among participants; however, most had fled ongoing war and conflict in their countries of origin, as well as political or religious persecution. All participants had temporary residency status except for one participant who was residing in Germany without a legal residence permit. A total of eight participants lived in private apartments, followed by seven participants who lived in refugee accommodation centers, and two participants who lived in shared flats. All participants identified as Muslims, and two identified as non-religious (or non-practicing) Muslims.

**Table 1:** Participant sociodemographics and clinical data.

**No**.	**Country of Origin**	**Religion**	**Months in Germany**	RHS Score^*^	PHQ-9 Score^*^	**Reasons for Migration**
1	Syria	Islam	15	21	13	War, individual, social
2	Syria	Islam	38	29	17	War, economic
3	Syria	Islam	29	32	17	War, individual
4	Syria	Islam	39	17	10	War, political, religious persecution
5	Syria	Islam	44	36	10	War, political, religious persecution
6	Iraq	Islam	8	37	15	Economic, social, political, and religious persecution
7	Iraq	Islam	21	38	17	War (Syria), political and religious persecution (Iraq)
8	Iraq	Islam	35	41	21	Political and religious persecution
9	Iraq	Islam	37	32	18	War, political situation, religious persecution. social
10	Syria	Islam	31	44	25	War, social
11	Iraq	Islam	47	43	23	War, political and religious persecution
12	Syria	Islam	39	20	19	War
13	Syria	Islam	24	35	16	War, economic, individual, political and religious persecution, social
14	Syria	Islam	31	17	7	Individual, social
15	Syria	Islam	21	24	10	War, individual
16	Syria	Islam	28	30	25	War, economic, social
17	Syria	Islam	44	33	21	War, individual, political and religious persecution

* *Scores of ≥12 on items 1–14 or ≥5 item 15 in the Refugee Health Screener-15 and “several days” or higher in a minimum of three responses the Patient Health Questionnaire-9 indicated psychological distress*.

**Table 2:** Summary of participant age and gender breakdown.

**Gender**	
Males	11
Females	6
**Age**	
20–29	8
30–39	4
40–49	5

### Themes

The main findings from the interviews were organized into four themes including, (I) faith-based coping during flight (II) changes in faith practices upon arrival (III) faith-based coping during integration, and (IV) advice for mental health providers. The first two themes capture an overview of general coping strategies in line with ongoing challenges and shifts in faith and faith practices experienced by participants upon arrival to Germany, and for the latter themes they provided examples of faith-based coping methods and how they can be incorporated into mental health care provided by non-Arab or non-Muslim mental health providers.

#### Faith-Based Coping During Flight

Participants provided examples of faith-based coping methods they utilized throughout their migration journey and after witnessing war and conflict in countries of origin. Among participants from Syria, these experiences were particularly acute since many had fled shortly following the onset of violence, experienced abrupt interruptions to schooling and livelihoods, witnessed the arrival of armed groups and were exposed to death or detention. Participants from Iraq described more protracted migration experiences, including living through multiple generations of war throughout childhood, experiencing long-term separation from family and children, and cited multiple experiences of displacement from Iraq.

Examples of faith-based coping methods were reported among participants who endured difficult or challenging displacement journeys, such as those who crossed multiple countries and borders to arrive to Germany, placing their families at risk in the process. One participant from Iraq shared:

*Once we took off by boat on the ocean, I asked God, “If I have a place in this world, let me and my entire family” to arrive. If you have written for someone in my family to drown, let me drown in their place. I hope I arrive to Germany in peace and safety. And if that anything was going to happen, it would be me instead of someone in my family*.

Two participants from Iraq also mentioned the importance of thanking God during or after the end of the journeys they endured by sea and foot to arrive to Germany:

*I said to myself, once I arrive, I will pray about 20 rakat* (supplications) *for God once we arrive to Germany. When I arrived to Germany, after about 10 days, I had a dream where God asked me, “Why did you not pray?” I felt someone was holding me accountable, why didn't you pray as promised? This was the first time something like this ever happened to me*.

#### Changes in Faith Practices Upon Arrival

Displacement to Germany resulted in processes of reflection among participants, who found that they had the opportunity, for the first time, to reflect on their personal beliefs and become more “open” to new perspectives and experiences that were not available in their country of origin. Upon displacement, participants reported that the cultural and religious disparity between Arab and German cultures made young refugee adults seek behaviors taboo to Islamic principles, such as drinking, smoking, and partying. On the other hand, participants reported that their displacement led to a greater understanding of individuals and religions outside of their own. This included exposures to churches, synagogues, as well as individuals who do not believe in God(s) or follow a specific faith. One participant from Syria stated:

*Of course, I became a lot more aware. I learned how to interact with people from different faiths and walks of life. I think this experience has made me a lot more aware. I do not think I will regret coming to Germany. On the contrary, I say, alhamdulilah* (thank God) *I arrived here and tried this. If I had stayed in Syria, I would have never experienced what it is like to be expatriated, to integrate in a new society, or with new religions, how to maintain yourself, culture and traditions in a new place, so I consider this [not only] an opportunity, but a nice chance*.

Another participant from Iraq stated:

*Things have changed here in Germany. I could go out whenever I want, I can do whatever I want. If I want to pray, I pray. If I want to drink, I drink. Whatever I want, I can do it. No one will tell me that this is against religion, or bad for the environment. I want my children to live their life without being judged*.

Most participants felt that the integration process was not contingent on or impeded by their faith. One participant from Syria stated that it was the responsibility of the refugee or migrant to acclimate, and that Germans were not responsible for acclimating to Arab or Muslim culture. While all participants interviewed identified as Muslims, two participants described themselves as “non-practicing” Muslims, noting changes that had occurred since they had arrived to Germany. One of these “non-practicing” participants, originally from Syria, used the example of seeing people from all walks of life on the metro to demonstrate his shift in thinking regarding religion:

*After a short time here [in Germany], you start thinking in a different way. You get on the metro, you start to see a lot of people – you ask why do these people think in a different way? Lots of incentive to ask yourself the question – “why am I this way? Why did I choose this religion [to follow]?” You then arrive to different convictions, you establish new convictions, depending on the circumstances*.

Other participants noted the consistency of their faith identity throughout their displacement and integration process, emphasizing that they felt no pressure or would not succumb to the pressure of changing their faith for the sake of integration. The following participant from Syria stated:

*If a German is to accept me, they will accept me as I am. I am not going to change so someone else can accept me. For those who are changing religiously, ethically, or culturally for others to accept them...I think that when Germans see someone like this [i.e. drinking alcohol in violation of their religious beliefs], then they will not respect them*.

Another participant from Syria shared how their faith has grown stronger since their arrival to Germany, particularly what they refer to as “the permanence” of God as a source of continuity, protection, and company in her new surroundings:

*In Syria, honestly, I was a bit more distracted with the world. I was living my normal life. Here, I am trusting of God, since I felt that my God is permanent, more so than people. In terms of my faith, God is everlasting and always there for me. Before, in Syria, I was always with my family, I had a routine, we were happy. All of a sudden, when you are alone...this is all from God. He permits you to travel safely, you come here, you walk by yourself, and you think of how much hardship there is in the world*.

When prompted to answer about changes in frequency of and commitment to faith practices, many participants cited having been more committed to practices in their country of origin than in Germany. For example, some participants reported praying less throughout the week, especially for those who worked full-time and could no longer attend Friday prayer or had limited access to an Arabic-speaking mosque or mosque of their Islamic sect. One participant from Syria stated:

*I feel this sort of hajiz* (barrier) *ever since I arrived to Germany. I miss the sound of the call to prayer* (athan). *I feel unable to pray and unmotivated to pray when I am here. Living in a Muslim country, like when I lived in Turkey, made a difference for me. It felt closer to home and reminded me of my faith practices more often. When I arrived to Germany, I developed averse feelings to religion and religious practices, which may be a result of my depression. In my worst moments, I am no longer motivated to seek help from God and feel demotivated from praying or practicing my faith*.

Some participants, mainly male, were also concerned about access to mosques and expressed distrust regarding religious leaders and mosques in Germany:

*I am finding some difficulties in maintaining prayer here. In Syria, I used to never miss a prayer, but not because I am less convinced [by my faith]. It is a shortage on my end. Near my house, there is no mosque near my house. The closest one is an hour away. My faith practice is inside my house, mainly*.

*One of the main reasons I do not go to the mosques in Germany is because there are no imams* (religious clerics) *in Germany like there were in Syria. Here, we do not know their backgrounds. They may be really good, but I do not know where they came from or the education they received to become an imam. In Syria, the imam was known by the village or city he lived in. Someone who is good, someone who is a hafiz* (memorizer of the Qur'an) –* you know that the society has nominated this person. Here, you do not know his background, and he could be influenced by foreign ideologies*.

#### Faith-Based Coping Methods to Address Distress During Integration

The majority of participants expressed that particular aspects of their faith and faith practices served as a positive source of comfort and reassurance throughout their mental distress and integration experience in Germany. Examples included attending religious services, making supplications, meeting other Muslims, and seeking help from a religious leader.

Some participants mentioned the importance of remembering and thinking of God as a means of coping with distress. One participant from Syria noted:

*Honestly, my faith in God is what keeps me going. I am convinced that the world is temporary… We know that there is a Hereafter, there is Heaven, there is something more beautiful, endless happiness, no anxiety, no sadness, no depression. This is something very comforting and brings me patience*.

Another participant from Iraq stated:

*I remember God without going to the mosque. While I am walking, I ask God to forgive me, to guide me, to release me, to keep me safe. A prayer is listened to no matter where you are, as long as it comes with an intention and a heart that is really broken or needs help*.

Other participants focused on the sense of calm they feel when reading Qur'an, praying, or supplicating. One participant from Syria shared:

*Religion helps those who understand it. Reading or hearing Quran cools* (calms) *the nerves. Sometimes I make supplication in order to ask for help, and I cry. You feel a weight on your body, that nothing in this world is worthwhile. When you read Quran or pray, you feel comfort all over your body, God makes you feel this sense of calm*.

Another participant from Iraq emphasized their reliance on prayer:

*Prayer makes me feel better because it makes me closer to God. He may forgive me, bless me, help me lead a path that is more different*.

More than one participant shared their thoughts on how faith-based methods of coping should be supplemented with medical treatment. The following was shared by a participant from Syria:

*I know people who use religion for everything. God said, “For everyone who tastes, there is medicine.” God says, “Ask for help [my worshipper], and I will help you,” if you want to seek treatment, and I will help you find it through your prayer. I will make the heart of the doctor feel for you, the pharmacist will help you. If I am sitting at home, and wait for God to treat me. God will not send us treatment in an envelope*.

Two participants from Syria, noted the lack of nearby mosques, which would have otherwise been a source of support when feeling distressed:

*If there was a mosque near my house, I think this would really help me. Sometimes depression and an overall mental health situation can impact one's mental health situation in a way that doesn't allow one to think realistically. The one thing that really helps me become stronger is religion, such as reading Quran or to pray (feel connected to God), makes me feel a sense of psychological well-being, to be honest*.

*Even if you have trouble in the real world, and you feel pressure, you go inside the mosque and start to cry. Once I leave, I feel like I am back to reality. Your negative thoughts start to escape you, your sadness. I start to feel much happier. I started to feel so depressed, and when I was hospitalized, I asked for a Quran and to visit a mosque. The translator came and he said he would bring me one as a gift*.

A few participants stated that they had sought help from a religious cleric. While some participants, particularly females, had positive experiences seeking support from religious clerics, one participant from Iraq noted a different experience:

*I tried to ask for help about my depressive symptoms, and the sheikh (religious cleric) told me to be make dua (supplication), to pray, to be patient, and to ask for forgiveness. I told him that I do not think I did anything wrong, that this depression that has existed for 4 years, it needs to be solved somehow*.

#### Advice for German Mental Health Providers

When participants were asked what they would like German (i.e., non-migrant) mental health providers about their cultural or spiritual backgrounds in order to optimize mental health treatment, a range of response was provided. In particular, the importance of the presence of a family and community for well-being were addressed, for example, by one Syrian participant:

*[It is] important that they [non-Arab health providers] understand Arab culture, such as where happiness comes from a societal perspective. For example, family is one of the most important pillars of happiness in Arab culture*.

Participants expressed their preference for Arabic-speaking mental health professionals (although, not necessarily Muslim) who could understand them directly, both in language, as well as the trauma they experienced before, during, and after their displacement. Put simply, by a participant from Iraq:

*I would like this person [the German mental health professional] to understand where I come from*.

The lack of a shared language for communication between patient and provider can also inhibit non-Arabic speaking mental health professionals from understanding culturally-specific manifestations of mental health conditions, such as a type of hair loss described by the same Iraqi participant, may be an explanation for particular mental health symptoms:

*For example, da' al tha'lab [in English: sudden hair loss or Alopecia areata] is a situation where you lose your hair as a result of fear or poor mental health. I had a year where I was dealing with this. The [German] doctor told me that this was a psychological condition. However, I know that it could be from fear (if you were robbed for example, someone robbed you) in addition to poor mental health*.

Another participant from Iraq also shared the need for empathy or a broader understanding of the trauma that was experienced by the client by the German or non-Arabic speaking mental health professional:

*If I told a German psychiatrist about the trauma I have endured, I would want them to be able to help with these experiences and to see it as a reality, not something that is fictional*.

Participants expressed the specific need for awareness among mental health professionals in Germany on specific aspects of their culture, religion, or traditional methods of coping, such as spiritual forms of mental health support, as described by a Syrian participant:

*A German psychiatrist would just treat your symptoms and give you a diagnosis. If someone who wants any kind of spiritual support, it might not be allowed. The doctor must really focus on religion. Because a renewal of the soul requires this sort of attention*.

## Discussion

This is one of few studies addressing faith-based coping methods among distressed Arabic-speaking refugees and asylum-seekers from Muslim-majority countries in Germany. Using a grounded theory approach, our analysis demonstrated a wide spectrum of definitions and interpretations of faith among Arabic-speaking refugees and asylum-seekers seeking mental health services, most of which had been shaped by challenging and often traumatic experiences before, during, and throughout their displacement and extending into the integration process. This was most explicitly demonstrated in the first three themes: (i) faith-based coping during flight, (ii) changes in faith practices upon arrival, and (iii) faith-based coping methods to address distress during integration.

Most participants in this sample had experienced significant challenges ahead of their arrival to Germany, including exposure to stressful events in Syria and Iraq before departure, multiple displacements and attempts to integrate into other host country contexts, detention and torture, and dangerous journeys by land or boat to arrive to Europe. Following arrival, participants cited social and economic barriers to integrating into German society, including difficulties learning the language, becoming accustomed to new culture, finding housing and employment, and the chronic uncertainty of what the future held for them. This had resulted in significant distress and negative mental health symptoms among those in the study sample, who had all decided to seek mental health treatment at the Charité Universitaetsmedizin-sponsored mental health clinic, where interviews took place.

Upon inquiry, our study found that the participants' dynamic relationship with their faith following their arrival to Germany played a direct role in how faith-based coping methods were or were not utilized when experiencing mental health symptoms. Most of those interviewed had only ever lived in Syria or Iraq, or had been displaced to Muslim-majority countries before their arrival to Germany. Particularly for male participants in this study, Germany provided a novel landscape for the exploration and interpretation of varying faith practices outside of own's own, and the integration process often involved a determination of which practices were helpful, or not so helpful, to their mental health and well-being. These trends are similar to findings from a recent study of Syrian refugees in the Netherlands, which demonstrated that levels of commitment to faith or religious practices influence coping strategies and overall feelings of integration (30). Those demonstrating a stronger commitment to faith were more likely to utilize faith-based coping strategies when seeking mental health services, including seeking support from religious leaders or local religious institutions.

We found notable differences in perspectives between male and female participants, female participants demonstrated of which demonstrated a greater reliance on faith-based coping mechanisms, including attending regular religious lectures and support groups in mosques, asking religious clerics for support with mental health symptoms, and reading Quran or praying in one's personal time. Male participants, on the other hand, expressed greater dissent than females with the religious infrastructures in Germany, including distrust of imams and particular religious bodies, lack of engagement with clerics for treatment and lower attendance of weekly (Friday) prayers. Nonetheless, and consistent with previous studies published on this topic (20, 26, 31) which highlight the intertwined nature of cultural and religious norms in these populations, faith was an enduring force in the lives of the majority of the Syrian and Iraqi refugee adults interviewed in this study, regardless of level of commitment to faith practices.

The fourth and final theme (iv) identified in this study included constructive advice from participants for German mental health providers, particularly providers who do not have a shared migrant background. These findings, which include a call for greater empathy and understanding of Syrian and Iraqi culture and faith practices, as well as specific ways of interpreting distress, could be particularly useful for German mental health providers engaging refugee and asylum-seeking populations. This includes the significance of family and community in the healing process, a common source of social support mechanism that may be absent for most refugees and asylum-seekers in Europe who are restricted from visiting family or have pending family reunification status. Positive faith-based coping strategies identified by participants to improve mental health outcomes, such help-seeking from religious leaders, reading Qur'an, remembering God, or making supplication can help inform service delivery by sharing these insights with mental health care providers in Germany (32). These perspectives also help identify themes of broader religious and social support in order to facilitate the integration of this population in their current context. The results of this study have implications for a variety of actors and stakeholders invested in facilitating both the short- and the long-term integration of such populations, including the need to develop culturally- and faith-sensitive interventions and to introduce cultural mediators to the clinical setting in order to facilitate the relationship between mental health provider and patient. Furthermore, results regarding positive faith-based coping methods demonstrate opportunities for local engagement from mosques and Islamic organizations with the Syrian, Iraqi, or broader Muslim refugee population, particularly in providing basic psychosocial support, mental health awareness, and expanding referrals to mental health professionals.

The cultural and context-specific interpretations of optimal mental healthcare by refugee communities provide insight on how non-profit organizations, faith-based organizations, and religious institutions can collaborate with mental health professionals to provide faith-based training and culturally-sensitive approaches to working with refugee populations as well as pose alternatives to the linguistic and cultural barriers posed by the German health system. This includes training for German mental health providers regarding the cultural and religious backgrounds of refugee clients they often provide care for, as well as overall sensitivity to the sociopolitical circumstances refugee clients escaped from ((33, 34). Religious clerics and spiritual leaders who are approached by refugee clients seeking faith-based treatment should also be trained to provide referrals to specialized mental health services for refugee populations (35).

An unanticipated finding was that many participants, when answering questions about their own faith identity and integration experiences, cited the experiences of others. This included current and former friends, members of their families, acquaintances, roommates, and a broader description of the refugee community at large (often identified as “the Syrians” or “the Arab community”). These generalizations provided a useful comparison for the participant, in order to either differentiate or state their similarity to this broader refugee community, particularly when describing shifts in their faith identities, their integration process, and their reliance on faith as a coping mechanism.

Furthermore, an important ethnographic consideration was the interchangeability of the concepts of religion, spirituality, cultures, and traditions that were utilized during the interviews. For example, expressing the extent of “religiosity” led to discussions regarding Syrian and Iraqi culture and traditions and how they differed extensively from those in Germany. The term “spirituality” was less understood by participants and is less referred to in the literature describing faith-based coping methods among Arab or Muslim populations (25, 36). Although there is limited information regarding the application of religious and spiritual healing methods for refugee populations who may have endured religious or ethnic persecution, there is significant literature on the application of these concepts in Islam and on Muslim populations broadly (37).

Due to the conceptual nature of the interviews, there were a number of limitations that emerged throughout the study.

The first limitation was that questions regarding faith-based coping often required an additional layer of explanation by the interviewer to each participant in order to clarify the intentions of the questions asked. This may have influenced answers given by participants following examples posed by the interviewer regarding faith-based coping, which included relying on prayer, reciting or reading scripture, or attending the mosque, in order to cope with particular mental health challenges. This was particularly the case given that these concepts, although designed using frameworks regarding faith-based coping in English, were inquired about and discussed in Arabic.

Another limitation of this study was the sensitive nature of the questions asked, particularly of participants who had faced religious persecution in their countries of origin. To address this issue, we aimed to clarify during interviews that these questions were aimed to support the improvement of mental health care and treatment provided to Arabic-speaking patients in Germany and in other Western contexts. This may have also led to answers that seemed more favorable or acceptable to the interviewer.

Lastly, all participants in this study were receiving treatment for their mental health symptoms and were therefore considered patients of the clinic in which the study was being conducted. This may have resulted in an overall wariness regarding what could be shared during the interviews, particularly criticisms of German or Arab mental health professionals who were currently working in the clinic. Furthermore, our sampling procedure included only Arabic-speaking individuals who demonstrated an interest in the topic of the study regarding faith-based coping and mostly represented individuals from Syria and Iraq. Future studies should attempt to represent the experiences of other refugee and asylum-seeking populations living in Germany and in Europe, more broadly.

## Conclusion

Overall, the results of this study demonstrate a variety of faith-based strategies for coping with displacement and the integration process among refugees and asylum-seeking populations from Arabic-speaking and Muslim-majority countries. The study also addresses changes in faith that this population may experience during integration and includes recommendations from refugees themselves to make mental healthcare services more culturally-sensitive. These findings also indicate the importance of understanding cultural- and faith-specific interpretations of mental health symptoms and subsequent actions for diagnosis and treatment of mental health conditions experienced by these populations. As European and North American countries remain top destinations for refugees and asylum-seekers, studies exploring culturally-specific mental health needs of refugees from Muslim-majority countries across Germany are critical to improving the quality of mental health services and in turn, facilitating social integration for these populations. The outcomes of this research could be beneficial for mental health professionals, non-governmental organizations, faith-based organizations, humanitarian aid agencies, and hospitals providing mental health and psychosocial support services to Arabic-speaking refugees in Western contexts. Future studies should take note of the perspectives of mental healthcare providers and other healthcare workers and mediators working with refugees throughout mental health clinics in Germany and in other Western contexts where a large majority of refugees from Arabic-speaking or Muslim-majority countries have been resettled.

## Data Availability Statement

The raw data supporting the conclusions of this article will be made available by the authors, without undue reservation.

## Ethics Statement

The studies involving human participants were reviewed and approved by Ethical Committee of the Charité Universitaetsmedizin Berlin. The patients/participants provided their written informed consent to participate in this study.

## Author Contributions

DR conceived of the study, collected the data, and coded the transcripts with inputs from MB throughout. DR performed the thematic analysis with feedback and input from MB and LW. DR wrote the manuscript with multiple revisions from MB, CK, DC, and LW. All authors contributed to the article and approved the submitted version.

## Conflict of Interest

The authors declare that the research was conducted in the absence of any commercial or financial relationships that could be construed as a potential conflict of interest. The handling editor declared a shared affiliation with the authors at time of review.

## References

[1] Silove D Ventevogel P Rees S. The contemporary refugee crisis: an overview of mental health challenges. World Psychiatry. (2017) 16:130–9. 10.1002/wps.20438 28498581 PMC5428192

[2] Elbert T Wilker S Schauer M Neuner F. Dissemination psychotherapeutischer Module für traumatisierte Geflüchtete. Der Nervenarzt. (2017) 88:26–33. 10.1007/s00115-016-0245-3 27853854

[3] Kaltenbach E Härdtner E Hermenau K Schauer M Elbert T. Efficient identification of mental health problems in refugees in Germany: the Refugee Health Screener. Eur J Psychotraumatol. (2017) 8:1389205. 10.1080/20008198.2017.1389205 29163869 PMC5687797

[4] Winkler J Brandl E Bretz H Heinz A Schouler-Ocak M. Psychische Symptombelastung bei Asylsuchenden in Abhängigkeit vom Aufenthaltsstatus. Psychiatr Praxis. (2018) 46:194. 10.1055/a-0806-3568 30541158

[5] Walther L Kröger H Tibubos AN von Scheve C Schupp J Tam Ta TM. Psychological distress among refugees in Germany: A cross-sectional analysis of individual and contextual risk factors and potential consequences for integration using a nationally representative survey. Br Med J Open. (2020) 10:e033658. 10.1136/bmjopen-2019-033658 32819926 PMC7440818

[6] Sandalio RN Life After Trauma: The Mental-Health Needs of Asylum Seekers in Europe. Washington, DC: Migration Policy Institute (2018).

[7] Georgiadou E Zbidat A Schmitt GM Erim Y. Prevalence of mental distress among Syrian refugees with residence permission in Germany: a registry-based study. Front Psychiatry. (2018) 9:393. 10.3389/fpsyt.2018.00393 30210373 PMC6121182

[8] Brandt L Henssler J Müller M Wall S Gabel D Heinz A. Risk of psychosis among refugees: a systematic review and meta-analysis. JAMA Psychiatry. (2019) 76:1133–40. 10.1001/jamapsychiatry.2019.1937 31411649 PMC6694397

[9] World Health Organization Mental Health Promotion and Mental Health Care in Refugees and Migrants: TECHNICAL guidance. Knowledge Hub on Health and Migration (2018). Available online at: https://www.euro.who.int/__data/assets/pdf_file/0004/386563/mental-health-eng.pdf (accessed May 18, 2020).

[10] UNHCR Global Trends: Forced Displacement in 2018. UNHCR (2018). Available online at: https://www.unhcr.org/en-us/statistics/unhcrstats/5d08d7ee7/unhcr-global-trends-2018.html (accessed December 31, 2020).

[11] Romei V Ehrenberg-Shannon B Maier-Borst H Chazan G How Well Have Germany's Refugees Integrated? Financial Times (2017). Available online at: https://www.ft.com/content/e1c069e0-872f-11e7-bf50-e1c239b45787 (accessed December 31, 2020).

[12] Siegart M Refugees' Religious Affiliation, Religious Practice, and Social Integration. Nuremberg: German Federal Office for Migration and Refugees (BAMF) (2020).

[13] Pew Research Center Europe's Growing Muslim Population. Pew Forum (2017). Available online at: https://www.pewforum.org/2017/11/29/europes-growing-muslim-population/ (accessed May 18, 2020).

[14] Falk R Refugees, Migrants and World Order. The Refugee Crisis and Religion. (2017). p. 23–34. London: Rowman and Littlefield International.

[15] Human Rights Watch European Union: Events of 2018. Human Rights Watch (2018). Available online at: https://www.hrw.org/world-report/2019/country-chapters/european-union (accessed May 18, 2020).

[16] Waagnvoorde R How Religion and secularism (don't) matter in the refugee crisis. In: Mavelli L Wilson EK editors. The Refugee Crisis and Religion. London: Rowman and Littlefield International (2017). p. 61–74.

[17] Su A Why Germany's New Muslims Go to the Mosque Less. The Atlantic (2017). Available online at: https://www.theatlantic.com/international/archive/2017/07/muslim-syrian-refugees-germany/534138/ (accessed May 18, 2020).

[18] Buber-Ennser I Goujon A Kohlenberger J Rengs B Multi-layered roles of religion among refugees arriving in Austria around 2015. Religions. (2018) 9:154 10.3390/rel9050154

[19] Böge K Karnouk C Hahn E Schneider F Habel U Banaschewski T. Mental health in refugees and asylum seekers (MEHIRA): study design and methodology of a prospective multicentre randomized controlled trail investigating the effects of a stepped and collaborative care model. Eur Arch Psychiatry Clin Neurosci. (2020) 270:95–106. 10.1007/s00406-019-00991-5 30796528

[20] Hassan G Kirmayer LJ Mekki-Berrada A Quosh C el Chammay R Deville-Stoetzel JB. Culture, Context and the Mental Health and Psychosocial Wellbeing of Syrians: A Review for Mental Health and Psychosocial Support Staff Working With Syrians Affected by Armed Conflict. Geneva: UNHCR (2015).

[21] Rathod S Kingdon D Pinninti N Turkington D Phiri P Cultural Adaptation of CBT for Serious Mental Illness: A Guide for Training and Practice. Hoboken, NJ: John Wiley and Sons (2015).

[22] Böge K Karnouk C Hahn E Demir Z Bajbouj M. On perceived stress and social support: depressive, anxiety and trauma-related symptoms in arabic-speaking refugees in Jordan and Germany. Front Public Health. (2020) 8:239. 10.3389/fpubh.2020.00239 32695739 PMC7338673

[23] Walker P Mazurana D Warren A Scarlett G Louis H The role of spirituality in humanitarian crisis survival and recovery. Sacred Aid: Faith and Humanitarianism. New York, NY: Oxford University Press (2012). p. 115–39.

[24] Pargament KI. The Psychology of Religion and Coping: Theory, Research, Practice. New York, NY: Guilford Press (2001).

[25] Hasan N Mitschke DB Ravi KE Exploring the role of faith in resettlement among Muslim Syrian refugees. J Religion Spirit Soc Work. (2018) 37:223–38. 10.1080/15426432.2018.1461045

[26] McMichael C ‘Everywhere is Allah's place’: Islam and the everyday life of Somali women in Melbourne, Australia. J Refugee Stud. (2002) 15:171–88. 10.1093/jrs/15.2.171

[27] Charité MEHIRA Health Service Research Available online at: https://www.charite.de/en/research/charite_research/research_projects/innovation_fund/mehira/ (accessed December 31, 2020).

[28] Charmaz K. ‘Discovering’chronic illness: using grounded theory. Soc Sci Med. (1990) 30:1161–72. 10.1016/0277-9536(90)90256-R 2360052

[29] Anandarajah G Hight E. Spirituality and medical practice: using the HOPE questions as a practical tool for spiritual assessment. Am Fam Phys. (2001) 63:81. 10.1016/s1443-8461(01)80044-7 11195773

[30] Safak A Kunuroglu FK van de Vijver F Yagmur K Acculturation of Syrian refugees in the Netherlands: religion as social identity and boundary marker. J Refugee Stud. (2020) 18–9. 10.1093/jrs/feaa020

[31] Grupp F Moro MR Nater UM Skandrani S Mewes R. ‘Only God can promise healing.’: help-seeking intentions and lay beliefs about cures for post-traumatic stress disorder among Sub-Saharan African asylum seekers in Germany. Eur J Psychotraumatol. (2019) 10:1684225. 10.1080/20008198.2019.1684225 31741719 PMC6844424

[32] Schweitzer R Van Wyk S Murray K Therapeutic practice with refugee clients: a qualitative study of therapist experience. Counsel Psychother Res. (2015) 15:109–18. 10.1002/capr.12018

[33] Hassan G Ventevogel P Jefee-Bahloul H Barkil-Oteo A Kirmayer LJ. Mental health and psychosocial wellbeing of Syrians affected by armed conflict. Epidemiol Psychiatric Sci. (2016) 25:129–41. 10.1017/S2045796016000044 26829998 PMC6998596

[34] Patel NSA Sreshta N The role of psychiatrists in the growing migrant and refugee crises. Am J Psychiatry Residents' J. (2017) 12:6–8. 10.1176/appi.ajp-rj.2017.120703

[35] Ali OM Milstein G Mental illness recognition and referral practices among imams in the United States. J Muslim Mental Health. (2012) 6:10 10.3998/jmmh.10381607.0006.202

[36] Ai AL Tice TN Huang B Ishisaka A Wartime faith-based reactions among traumatized Kosovar and Bosnian refugees in the United States. Mental Health Religion Cult. (2005) 8:291–308. 10.1080/13674670412331304357

[37] Pridmore S Pasha Mi Religion and spirituality: Psychiatry and Islam. Aust Psychiatry. (2004) 12:381–5. 10.1080/j.1440-1665.2004.02131.x 15715812

[38] Walther L Fuchs LM Schupp J von Scheve C. Living conditions and the mental health and well-being of refugees: evidence from a large-scale German survey. J Immigr Minor Health. (2020) 22:1–11. 10.1007/s10903-019-00968-5 31974927 PMC7441051

