# Supplementary material for: Faith-Based Coping Among Arabic-Speaking Refugees Seeking Mental Health Services in Berlin, Germany: An Exploratory Qualitative Study
Source: Front Psychiatry. 2021 Feb 1;12:595979. doi: 10.3389/fpsyt.2021.595979 (PMC7901912; doi:10.3389/fpsyt.2021.595979)
Supplement: Supplementary file 1 [file Table_1.DOCX]

***Appendix 1: Semi-structured interview guide***

**Introduction**

1. Where are you from?
2. How old are you?
3. Tell me about your family.
   1. Are you married?
   2. Do you have children?
4. Tell me about your living situation.
   1. Where do you live?
   2. Do you live alone?
5. Tell me about your migration to Germany.
   1. How old were you when you left your home country?
   2. When did you arrive?
   3. What other countries?
   4. Why did you leave?
   5. Do you wish to stay here?

**Challenges**

1. What are some difficulties you face in Germany? (Alternatively, challenges or stressors you are currently facing)
2. How is life different in Germany versus life in your home country?

**General Coping Methods**

1. What are your sources of hope, strength, comfort, and peace?
2. What do you hold on to during difficult times?

**Religious Background**

1. Are you part of a religious group?
   1. How important is this to you?
   2. What aspects of your religion are helpful or not so helpful to you?
2. Are you part of a religious spiritual community? Does it help you? How?
3. Do you have personal spiritual beliefs that are independent of organized religion? What are they? (e.g., prayer, meditation, reading scripture, attending religious services, listening to music, hiking, communing with nature)
4. How have the challenges (you mentioned above) you faced as a refugee/migrant to Germany strengthened or weakened your faith?

**Religious Coping**

1. For some people, their religious or spiritual beliefs act as a source of comfort and strength in dealing with life's ups and downs; is this true for you?
   1. If yes, go on.
   2. If no, Was it ever?
      1. If yes, what changed?)
2. Has being sick (or your current situation) affected your ability to do the things that usually help you spiritually?
   1. i.e. or affected your relationship with God?
3. Are you worried about any conflicts between your beliefs and your medical situation/care/decisions?
4. Would it be helpful for you to speak to a clinical chaplain/community spiritual leader? (Alternatively, have you spoken to one?)
5. Could you tell a little bit about your involvement with any local mosques?
6. How has your faith affected the way you live in the Germany as a refugee?
   1. If applicable, describe the faith experiences you had in any refugee camps or shelters.
   2. Could you tell about a particular experience as a refugee or migrant in which you had to use your religious beliefs to cope?
7. How often do you experience situations in which you have the feeling that God or something divine is present? (Huber & Huber, 2012)
8. What are some positive experiences that came as a result of your migrant experience?
   1. If any, could you relate those experiences to your religious practices and/or beliefs?
